# Deep proteogenomics; high throughput gene validation by multidimensional liquid chromatography and mass spectrometry of proteins from the fungal wheat pathogen *Stagonospora nodorum*

**DOI:** 10.1186/1471-2105-10-301

**Published:** 2009-09-22

**Authors:** Scott Bringans, James K Hane, Tammy Casey, Kar-Chun Tan, Richard Lipscombe, Peter S Solomon, Richard P Oliver

**Affiliations:** 1 Proteomics International, Perth, WA, Australia; 2 Australian Centre for Necrotrophic Fungal Pathogens, Murdoch University, Perth, WA, Australia; 3 Centre for Food and Genomic Medicine, Western Australian Institute for Medical Research, Perth, WA, Australia; 4 Plant Cell Biology, Research School of Biology, The Australian National University, Canberra 0200, ACT, Australia

## Abstract

**Background:**

*Stagonospora nodorum*, a fungal ascomycete in the class dothideomycetes, is a damaging pathogen of wheat. It is a model for necrotrophic fungi that cause necrotic symptoms via the interaction of multiple effector proteins with cultivar-specific receptors. A draft genome sequence and annotation was published in 2007. A second-pass gene prediction using a training set of 795 fully EST-supported genes predicted a total of 10762 version 2 nuclear-encoded genes, with an additional 5354 less reliable version 1 genes also retained.

**Results:**

In this study, we subjected soluble mycelial proteins to proteolysis followed by 2D LC MALDI-MS/MS. Comparison of the detected peptides with the gene models validated 2134 genes. 62% of these genes (1324) were not supported by prior EST evidence. Of the 2134 validated genes, all but 188 were version 2 annotations. Statistical analysis of the validated gene models revealed a preponderance of cytoplasmic and nuclear localised proteins, and proteins with intracellular-associated GO terms. These statistical associations are consistent with the source of the peptides used in the study. Comparison with a 6-frame translation of the *S. nodorum *genome assembly confirmed 905 existing gene annotations (including 119 not previously confirmed) and provided evidence supporting 144 genes with coding exon frameshift modifications, 604 genes with extensions of coding exons into annotated introns or untranslated regions (UTRs), 3 new gene annotations which were supported by tblastn to NR, and 44 potential new genes residing within un-assembled regions of the genome.

**Conclusion:**

We conclude that 2D LC MALDI-MS/MS is a powerful, rapid and economical tool to aid in the annotation of fungal genomic assemblies.

## Background

The primary goal of most, if not all, genome sequence projects is to elucidate the gene, and hence protein, content of the organism. The gene set is the key tool to elucidate the interesting biological aspects of the organism. The prediction of genes from assembled genomic data has traditionally relied on two types of data; sequenced transcripts and homology to gene sequences in related organisms. Based on these data, various *in silico *methods to predict gene models can be applied. Experience in intensively studied model organisms suggests that such methods still struggle to provide a reliable gene set. As more and more genome sequences of distantly related non-model species become available, the need for efficient, rapid and accurate methods of gene prediction becomes more and more pronounced.

Just as gene sequences can predict protein sequences, protein sequences can predict gene sequences [1]. Until recently, all methods to analyse peptide sequences in complex mixtures were too slow and too expensive to be considered a viable method of whole genome gene-model validation. Transcriptomic methods have been core to gene prediction as they can efficiently identify transcribed regions and define intron-exon boundaries. Proteomic methods focus on the translated regions of genes. They have been used to identify processed N and C termini of proteins and provide information about post-translational modifications [2-4]. Proteomics has also been used to measure the quantity of each protein because this is only poorly predicted by the quantity of transcript [3,5]. Proteomic analyses have hitherto been used to provide specific and complementary information about cellular function, but were not used as a primary method of gene annotation. Recent developments in proteomic techniques, using liquid chromatography, have begun to challenge the speed, cost and efficiency of gene validation by transcriptomics. A number of recent studies have reported the use of LC-based high-throughput proteomics to assist in refining genome annotation [6-9].

*Stagonospora (syn. Septoria or Phaeosphaeria) nodorum *is a major fungal pathogen of wheat in many parts of the world, causing Stagonospora nodorum blotch (SNB). In Western Australia it currently causes greater than $100 m losses per annum corresponding to 9% of the yield [10,11]. It is a member of the class dothideomycetes, a taxon that includes many important crop pathogen genera such as *Leptosphaeria, Mycosphaerella *and *Pyrenophora *[12]. A draft 37.1 Mbp genome assembly of a West Australian isolate (called SN15) was obtained in 2005. A total of 380,000 Sanger reads were obtained, corresponding to about 10× coverage. The reads were assembled into 496 contigs, 107 scaffolds and the mitochondrial genome. The total amount of gaps was estimated at 154 kb. A total of 15,455 reads were not included in the assembly [12].

The assembly was searched for genes using the Broad annotation pipeline. The pipeline used the then available EST data (just 317 manually curated transcripts) and conserved homology. This predicted 16,957 genes. This number was significantly higher than expected and so the annotation was checked by the acquisition of 21,503 EST sequences, principally from two libraries; an axenic library with oleate as carbon source and an *in planta *library made from heavily infected leaves with sporulating colonies. A total of 795 genes were manually annotated from comprehensive EST evidence. A second gene prediction run using Unveil predicted 10,762 nuclear and 14 mitochondrial version 2 (v2) genes [12,13]. One gene was present on the un-assembled reads. Of the nuclear genes, 2696 were supported by EST data. A further 5354 genes not supported by the second round of gene prediction were tentatively retained as version 1 (v1) gene models.

This uncertainty in gene content was hampering research efforts particularly because, in this case, homology-based gene prediction methods were unreliable. *S. nodorum *was the first dothideomycete to be sequenced and the nearest sequenced relative organisms are separated by 400 Mya [12]. A number of approaches to improving the confidence in gene models could be envisaged. In this study, we analysed soluble mycelial proteins using 2D LC-MS/MS to generate a library of mass spectra. The data were used to verify our current gene prediction models. In addition, we generated six-frame translations of the assembled and un-assembled genome sequences to facilitate the discovery of unidentified genes and correction of current coding exon boundaries and frame assignment. To our knowledge, this study is the first that describes the extensive use of high-throughput proteomics in assisting gene annotation of a phytopathogenic fungus. The data supports 2253 genes, a number comparable to that supported by an extensive EST project. It also highlighted many potential gene model problems and identified new gene candidates.

## Methods

### Growth and Maintenance of *Stagonospora nodorum*

*S. nodorum *SN15 and *gna1 *strains were maintained on CZV8CS agar as previously described [14]. These two strains were chosen as part of a relative quantitation analysis coupled to this proteome mapping experiment. For proteomic analysis, 100 mg of fungal mycelia were inoculated into minimal medium broth supplemented with 25 mM glucose as the sole carbon source. The fungi were grown to a vegetative state by incubation at 22°C with shaking at 150 rpm for three days. Vegetative mycelia were harvested via cheesecloth filtration and freeze-dried overnight.

### Protein Extraction

Soluble intracellular proteins were extracted from freeze-dried mycelia as previously described [2]. Briefly, freeze-dried mycelia were mechanically broken with a cooled mortar and pestle and proteins were solubilised with 10 mM Tris-Cl (pH 7.5). The crude homogenate was collected and centrifuged at 20,000 *g *for 15 min at 4°C. The resulting supernatant was retained and treated with nucleases to remove nucleic acids. All protein samples were checked via SDS-PAGE to ensure that proteolysis was minimal during sample preparation (data not shown).

### Sample Preparation

Proteins from SN15 and *gna1-35 *strains were precipitated individually by adding five volumes of acetone, incubating for 1 hour at -20°C and pulse centrifuging for 5-10 seconds. The protein pellets were resuspended in 0.5 M triethylammonium bicarbonate (TEAB) (pH 8.5) before reduction and alkylation according to the iTRAQ protocol (Applied Biosystems, Foster City, CA, USA). Samples were centrifuged at 13,000 *g *for 10 min at room temperature before the supernatant was removed and assayed for protein concentration (Bio-Rad protein assay kit, Hercules, CA, USA). A total of 55 μg of each sample was digested overnight with 5.5 μg trypsin at 37°C. Each digest was desalted on a Strata-X 33 μm polymeric reverse phase column (Phenomenex, Torrance, CA, USA) and dried. The entire experiment was performed in triplicate (including the generation of ground mycelia).

### Strong Cation Exchange Chromatography

Dried peptides were dissolved in 70 μl of 2% acetonitrile and 0.05% trifluoroacetic acid (TFA) and separated by strong cation exchange chromatography on an Agilent 1100 HPLC system (Agilent Technologies, Palo Alto, CA, USA) using a PolySulfoethyl column (4.6 × 100 mm, 5 μm, 300 Å, Nest Group, Southborough, MA, USA). Peptides were eluted with a linear gradient of Buffer B (1 M KCl, 10% acetonitrile and 10 mM KH_2_PO_4_, pH 3). A total of 37 fractions were collected, pooled into 8 fractions, desalted, dried and resuspended in 20 μl of 2% acetonitrile and 0.05% TFA.

### Reverse Phase Nano LC MALDI-MS/MS

Peptides were separated on a C18 PepMap100, 3 μm column (LC Packings, Sunnyvale, CA, USA) with a gradient of acetonitrile in 0.1% formic acid using the Ultimate 3000 nano HPLC system (LC Packings-Dionex, Sunnyvale, CA, USA). The eluent was mixed with matrix solution (5 mg/ml α-cyano-4-hydroxycinnamic acid) and spotted onto a 384 well Opti-TOF plate (Applied Biosystems, Framingham, MA, USA) using a Probot Micro Fraction Collector (LC Packings, San Francisco, CA, USA).

Peptides were analysed on a 4800 MALDI-TOF/TOF mass spectrometer (Applied Biosystems, Framingham, MA, USA) operated in reflector positive mode. MS data were acquired over a mass range of 800-4000 *m/z *and for each spectrum a total of 400 shots were accumulated. A job-wide interpretation method selected the 20 most intense precursor ions above a signal/noise ratio of 20 from each spectrum for MS/MS acquisition but only in the spot where their intensity was at its peak. MS/MS spectra were acquired with 4000 laser shots per selected ion with a mass range of 60 to the precursor ion -20.

### Data Analysis

Mass spectral data from all three biological replicates were combined and analysed using the Mascot sequence matching software (Matrix Science, Boston, USA) with the support of the facilities at the Australian Proteomics Computational Facility (Victoria, Australia). Search parameters were: Enzyme, Trypsin; Max missed cleavages, 1; Fixed modifications, iTRAQ4plex (K), iTRAQ4plex(N-term), Methylthio(C); Variable modifications, Oxidation(M); Peptide tol, 0.6 Da; MS/MS tol, 0.6 Da. The MOWSE algorithm (MudPIT scoring) of Mascot was used to score the significance of peptide/protein matches with p < 0.05 for each protein identification. Four protein datasets were constructed for proteogenomic screening: the combination of version 1 and 2 proteins as defined from annotation of the SN15 genome sequence [12]; a between-stop codon 6-frame translation of the *S. nodorum *genome assembly; 6-frame translated, CAP3-generated [15] contigs of un-assembled reads of the *S. nodorum *assembly, and; 6-frame translated singleton un-assembled reads which did not assemble into contigs via CAP3. All 6-frame open reading frames (ORFs) were subject to a 10 amino acid minimum length threshold.

For the purpose of false discovery rate (FDR) calculation, randomised sequences from the version 1 and 2 proteins and the 6-frame translated assembly protein datasets were generated as Mascot decoy databases [16] (as detailed at http://www.matrixscience.com/help/decoy_help.html).

### Characterisation of peptide-supported genes

Peptide supported genes were analysed for abundance of assigned gene ontology (GO) terms [12]. Gene counts for GO terms were compared between peptide supported and unsupported genes via Fisher's exact test. A p-value threshold of 0.05 was imposed to determine significance. Gene counts for SignalP [17] and WolfPsort [18] cellular location predictions and relative molecular mass predictions were also compared by this method.

### De novo proteogenomics

MudPIT-filtered peptide matches to the 6-frame translated assembly were mapped back to their genomic location. Peptides mapping in the same orientation with either overlapping genomic coordinates or within the proximity of 200 bp were combined as peptide clusters (referred to herein as peptide clusters). The purpose of peptide cluster formation was merely to reduce the redundancy in the peptide data to aid in the interpretation of subsequent comparisons with annotated gene features, therefore clusters with a single peptide were retained. Individual peptides and peptide clusters were compared for overlap and proximity within 200 bp to *S. nodorum *version 1 and 2 genes.

**Figure 1:**
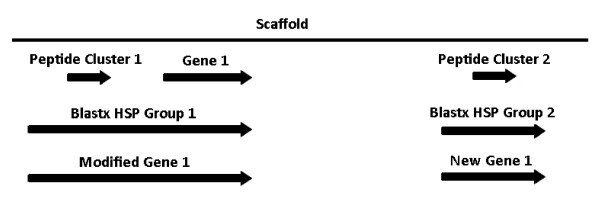
**Peptide clusters which did not confirm an existing gene model or conflict with an existing gene model in the opposing orientation were compared to grouped blastx HSPs from related dothideomycete proteins**. Some of these peptide clusters were linked back to existing gene models as illustrated by Peptide Cluster 1 and Gene 1, which share a homology relationship with Blastx HSP group 1. This provides strong evidence for the reannotation of Gene 1 to become Modified Gene 1. Other peptide clusters could not be linked to existing gene annotations such as Peptide Cluster 2, which provides evidence for the creation of New Gene 2.

Potential homologs to *S. nodorum *SN15 genes were detected by tblastn comparison of the genome assembly with the proteomes of the dothideomycete fungi *Leptosphaeria maculans*, *Pyrenophora tritici-repentis*, *Cochliobolus heterostrophus*, *Alternaria brassicicola*, *Mycosphaerella graminicola *and *Mycosphaerella fijiensis*. Tblastn high-scoring pairs (HSPs) were grouped according to hit, but also subject to additional criteria: best HSP e-value < 1e-10; individual HSP e-values < 1e-5; HSPs mapped on *S. nodorum *genome no further than 2 kb apart or split into sub-groups each subject to the previous criteria. Grouped HSP sequence coordinates were compared to both peptide cluster and annotated gene coordinates on the *S. nodorum *genome assembly for overlap (Figure 1). By this method we detected peptide clusters which could be linked back to a nearby gene model through a shared homolog or peptide clusters representing potential new gene annotations with homology support.

## Results and Discussion

### Gene models confirmed by Mascot analysis of the existing gene model database

**Figure 2:**
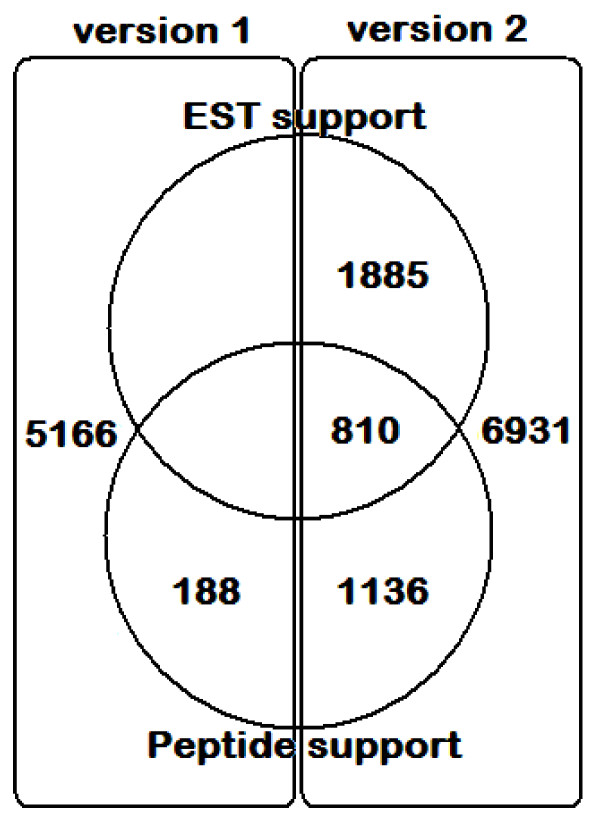
**Comparison of *S. nodorum *annotated gene versions and confirmation by either MASCOT peptide matching or EST alignment**. Version 2 genes are derived from EST alignments and a second round of EST-trained gene predictions. Version 2 genes and are considered to be more reliable than the remaining tentative version 1 gene annotations.

Comparison of the detected peptides against a database built from both v1 and v2 predictions of SN15 genes matched a total of 2134 gene sequences with high confidence (Tables 1, 2 and 3). Of these, all but 188 were v2 genes (Figure 2; new and modified genes are listed in Additional Files 1 and 2). The results indicate the greater reliability of the v2 prediction. The proteomic analysis matched 1324 genes that were not directly supported by any of the 21,503 EST sequences (Figure 2). This clearly shows that proteomics targets a complementary set of gene products to transcriptomic approaches. The false discovery rate (FDR) for the mass spectra matched against *S. nodorum *proteins was determined to be 13% by the Mascot decoy method. While this is relatively high, the purpose of this study was the discovery of genes not detectable by conventional techniques. Hence we favoured sensitivity over accuracy.

**Table 1:** Sub-cellular localisation predictions using WolfPsort of proteins over-represented within the Mascot peptide supported gene annotations of *S. nodorum*.^1^

Localisation	Peptide supported genes (all)	Peptide supported genes (v1)	Peptide supported genes (v2)	Unsupported genes	Expected genes	Total Genes
**cytoplasm**	605	24	581	2118	361	2723
**cytoplasm/nucleus**	87	7	80	491	77	578
**other**	1442	157	1285	11346	1094	12788
**unassigned**	0	0	0	27	4	27
**TOTAL**	**2134**	**188**	**1946**	**13982**	**2136**	**16116**

^1^Numbers of peptide supported genes in each category were compared to expected counts from a random sampling of the whole genome via Fisher's exact test at a significance threshold of <0.05.

**Table 2:** Sub-cellular localisation predictions using SignalP of proteins over-represented within the Mascot peptide supported gene annotations of *S. nodorum*.^1^

	Peptide supported genes (all)	Peptide supported genes (v1)	Peptide supported genes (v2)	Unsupported genes	Expected genes	Total Genes
**non-secretory protein**	1847	163	1684	11259	1773	13106
**signal anchor OR signal peptide**	287	25	262	2723	436	3010
**TOTAL**	**2134**	**188**	**1946**	**13982**	**2209**	**16116**

^1^Numbers of peptide supported genes in each category were compared to expected counts from a random sampling of the whole genome via Fisher's exact test at a significance threshold of <0.05.

**Table 3:** Relative molecular masses of predicted proteins significantly over-represented within the Mascot peptide supported gene annotations of *S. nodorum*.^1^

	Peptide supported genes (all)	Peptide supported genes (v1)	Peptide supported genes (v2)	Unsupported genes	Expected genes	Total Genes
**0 to 20 kDa**	284	60	224	4495	633	4779
**20 to 100 kDa**	1536	121	1415	8816	1371	10352
**100 to 500 kDa**	310	7	303	669	130	979
**>500 kDa**	4	0	4	2	1	6
**TOTAL**	**2134**	**188**	**1946**	**13982**	**2135**	**16116**

^1^Numbers of peptide supported genes in each category were compared to expected counts from a random sampling of the whole genome via Fisher's exact test at a significance threshold of <0.05.

The genes validated by the 2D-LC-MS/MS procedure were searched for features that were overrepresented compared to the total set of predicted genes. Peptide-supported genes predicted by WolfPsort to be localised in the cytoplasm were over-represented whereas cytoskeletal, extracellular, mitochondrial, peroxisomal and plasma membrane samples were under-represented. Peptides with predicted masses greater than 20 kDa were over-represented. Detailed analyses of GO-terms over- and under-represented are given in Additional Files 3 and 4. These analyses are consistent with the source of the peptides used in this analysis in which only soluble, intracellular proteins were isolated and analysed. The data indicates that using extracellular, membrane and cell-wall material would significantly increase the number of proteins detected. If a similar proportion of protein detection was achieved for these proteins as was for the soluble, cytoplasmic proteins, the number of extra genes detected would be about 1400.

### Gene models confirmed by Mascot analysis of the 6-frame translated genome database

The proteomic data can also be used to search for gene models with errors such as inaccurate exon-intron boundaries and missed or superfluous exons as well as entirely unsuspected genes. We approached this by mapping peptides onto the 6-frame translation of the nuclear genome assembly. The coordinates of overlapping or nearby mapped peptides were amalgamated into peptide clusters Peptide clusters were defined so that internal gaps between constituent peptides of up to 200 bp were permitted, which is about four times the average intron size in *S. nodorum *[12].

Initially the mapped-peptides were compared to existing annotated coding exons (Table 4). Because of the uncertainty in defining the ends of exons, hits within 200 bp were also scrutinised. A total of 12947 spectra mapped to the genome assembly. Of these 11635 mapped within coding exons, 323 mapped within 200 bp of a coding exon and 989 mapped distantly from known genes.

**Table 4:** Summary of 12947 6-frame translated genome-mapped peptides and 1840 peptide clusters corresponding to annotated gene features categorised by either direct overlap or close proximity (within 200 bp).

Match Type	Overlap	Within 200 bp	No match	Total
**Peptide-CDS**^1^	11635	323	989	**12947**
**Peptide cluster-CDS**	1520	54	266	**1840**

^1 ^CDS features are coding exons (gene sequence from translation start to stop, excluding introns). The number of proximal peptide and peptide cluster matches is greatly reduced in whole gene comparisons relative to CDS matches, indicating significant mis-annotations in EST-supported UTR regions and/or intron regions.

A similar analysis was also carried out by comparing peptide clusters to the genome assembly (Figure 1). A total of 1840 peptide clusters were analysed. Of these, 1520 mapped within coding exons, 54 mapped within 200 bp and 266 mapped distantly from known genes. The 1520 confirmed peptide clusters correspond to 905 genes (Table 5). Of these, 119 were not identified by the previous Mascot analysis of the gene models, bringing the total of confirmed genes to 2253 (Additional File 2). The genes identified by 6-frame analysis corresponded overwhelmingly to v2 genes. Only 41 were v1 genes and 30 of these exhibited potential conflicts with the current gene model. All genes with EST support had previously been designated v2. In these cases, it was possible to define un-translated (UTR) regions, within both introns and terminal exons. The v2 confirmed genes included 300 without conflict and 564 with potential exon conflicts, divided between genes with (355) and without (209) EST support.

**Table 5:** Counts of *S. nodorum *version 1 and 2 gene annotations matching 6-frame translated genome-mapped peptide clusters.^1^

Annotation version	Confirmed no conflict	UTR/intron conflict (EST support)	UTR/intron conflict (no EST support)	No-match	Total
**1**	11	0	30	5313	5354
**2**	300	355	209	9898	10762

^1^Un-translated region (UTR)/intron conflicts were determined based on peptide matches outside of coding exon regions but within either 200 bp or within the boundaries of known UTRs. UTRs were known for genes for which EST alignments were available, where UTR regions were defined as EST-aligned regions not corresponding to coding exons or introns. 41% of peptide-supported version 2 (reliable) genes with UTR regions confirmed by EST support have suspected UTR/intron conflicts (355 out of 864).

These analyses so far have merely mapped 6-frame translated genome-mapped peptide or peptide cluster coordinates to exon or gene coordinates. Next we considered the predicted open reading frames of the exons and the individual mapped peptides overlapping annotated coding exons (Table 6). Reassuringly, 11224 peptides matched exactly to the predicted frame whereas 482 entirely matched to a different frame. When analysed by gene, 715 were fully confirmed; of these 13 were v1 genes and 702 were v2. In a total of 144 genes, there were frame mismatches detected by all (86) or some (58) of the supporting peptides.

**Table 6:** Summary of frame conflicts within coding-exon (CDS) annotations confirmed by overlapping 6-frame translated genome-mapped peptides.

TOTAL peptide-CDS matches in frame	11224
TOTAL peptide-CDS matches out of frame	482
Genes with all peptide matches to CDS in frame	715
Version 1	13
Version 2	702
Genes with all peptide matches to CDS out of frame	86
Version 1	10
Version 2	76
Genes with peptide matches to CDS both in and out of frame	58
Version 1	1
Version 2	57

The majority of peptide matches agree with current coding-exon frames, however there are 144 (86+58) gene annotations requiring frame reassessment.

### Gene models identified by Mascot analysis of the 6 frame translated un-assembled read database

Finally, the mass spectra were compared to the collection of un-assembled DNA reads. The 15,455 reads were re-assessed for overlapping sequence missed during the first genome assembly by clustering into contigs using CAP3 [15]. 4616 reads were clustered into 939 contigs, with 10,839 singleton reads remaining. 423 contigs and 651 singleton DNA reads matched peptides that were detected by MS.

### Sifting new and modified gene models by homology criteria

The probabilistic nature of the matching of the peptides to the genome is reflected in the false discovery rate, estimated at 13% for the matches against existing gene models. Thus not all the gene confirmations, the conflicts with existing models or the potential new genes can be expected to survive scrutiny.

**Figure 3:**
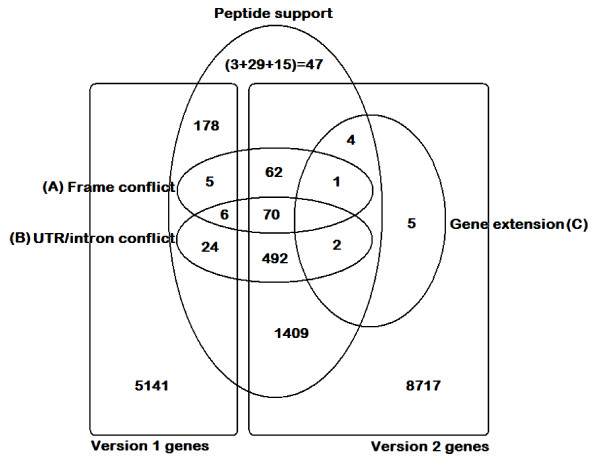
**Summary of version 1 and 2 *S. nodorum *genes confirmed and modified by peptide support (either by conventional protein database or by 6-frame genome translation-derived peptide matches)**. Genes were identified as candidates for re-annotation if the 6-frame translated genome-matched peptides indicated: conflicts in annotated coding-exons open reading frames (A); peptide-genome matches residing within annotated introns or untranslated regions (UTRs) (B) or; peptide-genome matches matched to the genome which could be linked back to a gene model via tblastn homology between the genome sequence and selected dothideomycete genomes (C). 47 new gene candidates were identified by a multiple methods: 3 peptide clusters which could not be linked to an existing gene annotation via a tblastn homolog; 29 unassembled read-contigs matching dothideomycete proteins via blastx but not matching *S. nodorum *proteins and; 15 unassembled read-singletons matching dothideomycete proteins via blastx but not matching *S. nodorum *proteins.

Overall, these data suggest the confirmation of 2254 previously defined v1 and v2 genes (Figure 3). Of these, about 594 (355 + 30 + 209, Table 5) are identified as having questionable exon boundaries and 144 (86 + 58) as having questionable frame assignments (Table 6). Confirmation or rejection of the new gene models will require gene-by-gene sifting of the evidence; however this analysis remains an effective method of highlighting gene annotations with potential problems.

Analysis of the assembly identified a potential 266 peptide clusters that mapped distant from known genes and represent candidate novel genes. Assessment of the unassembled reads identified a further 1074 (423+ 651) candidate genes (Tables 7 and 8).

**Table 7:** Summary of the 266 6-frame translated genome-mapped peptide clusters^1 ^not confirming existing *S. nodorum *CDS annotations by either overlap or proximity within 200 bp.

In conflicting orientation with existing gene annotation	113
No conflict, no supporting evidence	135
Overlaps genomic tblastn hit	18
Genomic tblastn hit links to an existing gene	15
Genomic tblastn hit	3

^1 ^Peptide clusters were assessed for orientation conflicts with genes in the opposing strand and for overlap with grouped regions of tblastn homology to related dothideomycete genomes (*L. maculans*, *P. tritici-repentis*, *C. heterostrophus*, *A. brassisicola*, *M. graminicola *and *M. fijiensis*).

**Table 8:** Summary of 6-frame translated unassembled reads supported by MASCOT peptides.

**Unassembled Read Contigs**	**939**
with peptide support	423
with blastx hit to dothideomycetes	270
Hits *S. nodorum *gene	241
does not hit *S. nodorum *gene	29
Without blastx hit to dothideomycetes	153
without peptide support	516
**Unassembled Read Singletons**	**10839**
with peptide support	651
with blastx hit to dothideomycetes	437
hits *S. nodorum *gene	422
does not hit *S. nodorum *gene	15
Without blastx hit to dothideomycetes	214
without peptide support	10188

15455 reads that were not included in the main genome assembly of *S. nodorum *were re-assessed for overlap. 4616 reads were clustered into 939 contigs, with 10839 singleton reads remaining.

To sift through this large number of new gene candidates, we applied two tests. First we discarded gene candidates that mapped to known genes in the opposing orientation. This removed 113 of the 266 peptide clusters matching the assembly. As the genomes of several dothideomycetes have been released in the last three years, we were able to compare the predicted new genes to the predicted proteomes of these related organisms. Overall 68% (10899/16116) of *S. nodorum *genes (and 86.4% of v2 (9299/10762) genes) have a homolog among these related species. Only 18 peptide clusters (12%) corresponded to significant tblastn hits between dothideomycete proteins and the genome assembly. Of these, 15 matched known *S. nodorum *genes (Additional File 2A) and six of these corresponded to a single gene (SNOG_01477). The three other peptide clusters correspond to potentially novel genes (Additional File 2B). In the case of the 1074 candidate genes on the un-assembled reads, 707 (270 + 437) significantly matched via blastx to dothideomycete proteins of which 663 (241 + 422) hit existing *S. nodorum *genes. The remaining 44 (29 + 15) genes are dominated by transposon-related genes (as would be expected for the repeat-dominated un-assembled reads) but also include several metabolically and structurally critical gene functions (Table 8 and Additional File 2C).

## Conclusion

The flood of genome sequences that are resulting from the wave of "next-generation" sequencing technologies demands the development of time and cost-efficient methods of genome annotation. Annotation pipelines utilising transcriptomic data will remain the first choice option in many cases but the results presented here show that proteomics based on LC and tandem TOF approaches can efficiently complement transcriptomic-based annotation. The number of genes confirmed by EST analysis (2696) and proteomics (2253) are comparable both in terms of experimental time and equipment and consumables costs. The confirmation of existing gene models by proteomics is computationally straightforward as is the matching of spectra to 6-frame translated genome databases. Merging and resolution of multiple datasets of differing evidence levels is more complicated. In this paper, we have developed an annotation protocol based on defining peptide clusters and comparing first their coordinates to existing genes and then their sequences to genes from related organisms. Building upon this, we have created a pipeline that highlights potential problems with existing genes as well as new genes. This approach does not replace the need for manual annotation but reduces the scale of the task whilst providing an additional layer of evidence for gene annotation refinement.

## Authors' contributions

KCT performed fungal culture, protein extraction and sample preparation techniques. PSS and RPO devised the experimental plan and provided intellectual input. SB, TC and RL performed mass-spectrometry analyses. JKH performed the bioinformatic analyses. JKH and RPO wrote the manuscript and RPO, PSS, KCT, JKH and RL revised the manuscript. All authors have read and approved the final manuscript.

## Supplementary Material

Additional file 1 Summary of the potentially new and modified genes identified by 6-frame proteogenomics. 18 6-frame translated genome-matching Peptide clusters not supporting an existing gene annotation with tblastn homology evidence supported the modification (by extension and/or merging) of 12 existing gene annotations (A) or the creation of a new gene annotation (B). 6 of the 15 Peptide clusters linked to existing gene annotations corresponded a single gene, SNOG_01477. 29 contigs of unassembled reads had a blastx hit to a dothideomycete genome but no similarity with *S. nodorum *annotated genes (C). These represent potential new genes that were excluded from the *S. nodorum *genome due to assembly errors. A further 15 unassembled reads which did not form contigs also had a blastx hit to a dothideomycete genome but no similarity with *S. nodorum *annotated genes (D). These represent a less reliable set of potential new genes excluded from the main genome assembly. % Hit aligned is the percentage of the length of the best blastp hit subsequently globally aligned via the Needleman-Wunsch algorithm that aligns to the corresponding *S. nodorum *protein. % Identity is the percentage of identical amino acids contained within this alignment, whereas % Similarity is the percentage of amino acids with similar properties. Click here for file

Additional file 2 Summary tables of supporting evidence for peptide-supported genes and coordinate data for 6-frame translated genome-mapped peptides and peptide clusters. Click here for file

Additional file 3 Summary of gene ontology (GO) terms over and under represented in peptide supported *S. nodorum *genes relative to a random sampling of the whole genome of *S. nodorum*. Significance of representation was determined via Fisher's exact test, subject to a p-value threshold of 0.05. Click here for file

Additional file 4 Summary of gene ontology (GO) terms over and under represented in peptide supported *S. nodorum *genes relative to genes supported by EST alignments. Significance of representation was determined via Fisher's exact test, subject to a p-value threshold of 0.05. Click here for file
