# Supplementary material for: Deep proteogenomics; high throughput gene validation by multidimensional liquid chromatography and mass spectrometry of proteins from the fungal wheat pathogen Stagonospora nodorum
Source: BMC Bioinformatics. 2009 Sep 22;10:301. doi: 10.1186/1471-2105-10-301 (PMC2753851; doi:10.1186/1471-2105-10-301)
Supplement: Additional file 1 — Summary of the potentially new and modified genes identified by 6-frame proteogenomics. 18 6-frame translated genome-matching Peptide clusters not supporting an existing gene annotation with tblastn homology evidence supported the modification (by extension and/or merging) of 12 existing gene annotations (A) or the creation of a new gene annotation (B). 6 of the 15 Peptide clusters linked to existing gene annotations corresponded a single gene, SNOG_01477. 29 contigs of unassembled reads had a blastx hit to a dothideomycete genome but no similarity with S. nodorum annotated genes (C). These represent potential new genes that were excluded from the S. nodorum genome due to assembly errors. A further 15 unassembled reads which did not form contigs also had a blastx hit to a dothideomycete genome but no similarity with S. nodorum annotated genes (D). These represent a less reliable set of potential new genes excluded from the main genome assembly. % Hit aligned is the percentage of the length of the best blastp hit subsequently globally aligned via the Needleman-Wunsch algorithm that aligns to the corresponding S. nodorum protein. % Identity is the percentage of identical amino acids contained within this alignment, whereas % Similarity is the percentage of amino acids with similar properties. [file 1471-2105-10-301-S1.PDF]

Additional file 1: Summary of the potentially new and modified genes identified by 6-frame proteogenomics. 18 6-frame translated genome-matching Peptide clusters not supporting an existing gene annotation with tblastn homology evidence supported the modification (by extension and/or merging) of 12 existing gene annotations (A) or the creation of a new gene annotation (B). 6 of the 15 Peptide clusters linked to existing gene annotations corresponded a single gene, SNOG_01477. 29 contigs of unassembled reads had a blastx hit to a dothideomycete genome but no similarity with *S. nodorum* annotated genes (C). These represent potential new genes that were excluded from the *S. nodorum* genome due to assembly errors. A further 15 unassembled reads which did not form contigs also had a blastx hit to a dothideomycete genome but no similarity with *S. nodorum* annotated genes (D). These represent a less reliable set of potential new genes excluded from the main genome assembly. % Hit aligned is the percentage of the length of the best blastp hit subsequently globally aligned via the Needleman-Wunsch algorithm that aligns to the corresponding *S. nodorum* protein. % Identity is the percentage of identical amino acids contained within this alignment, whereas % Similarity is the percentage of amino acids with similar properties.

(A) Modified (extended) genes

Peptide Cluster	Corresponding existing gene annotations	Accession	Best informative blastp match to *S. nodorum* gene(s)	Organism	Accession	% Hit aligned	% Identity	% Similarity
PEPTIDE_CLUSTER_98	SNOG_00928	EAT92423.2	enhancer of zeste homolog 1 (Drosophila), isoform CRA_a	*Mus Musculus*	EDL03898.1	27.7%	41.0%	54.6%
PEPTIDE_CLUSTER_189	SNOG_01477	EAT91126.2	glutamate synthase precursor	*Pyrenophora tritici-repentis* Pt-1C-BFP	EDU46825.1	43.5%	93.9%	96.9%
PEPTIDE_CLUSTER_190	SNOG_01477	EAT91126.2	glutamate synthase precursor	*Pyrenophora tritici-repentis* Pt-1C-BFP	EDU46825.1	43.5%	93.9%	96.9%
PEPTIDE_CLUSTER_191	SNOG_01477	EAT91126.2	glutamate synthase precursor	*Pyrenophora tritici-repentis* Pt-1C-BFP	EDU46825.1	43.5%	93.9%	96.9%
PEPTIDE_CLUSTER_192	SNOG_01477	EAT91126.2	glutamate synthase precursor	*Pyrenophora tritici-repentis* Pt-1C-BFP	EDU46825.1	43.5%	93.9%	96.9%
PEPTIDE_CLUSTER_193	SNOG_01477	EAT91126.2	glutamate synthase precursor	*Pyrenophora tritici-repentis* Pt-1C-BFP	EDU46825.1	43.5%	93.9%	96.9%
PEPTIDE_CLUST	SNOG_01477	EAT91126.2	glutamate synthase precursor	*Pyrenophora tritici-*	EDU46825.1	43.5%	93.9%	96.9%

ER_194				*repentis* Pt-1C-BFP				
PEPTIDE_CLUSTER_321	SNOG_02518	EAT90730.2	transcription factor TFIIIB complex subunit Brf1, putative	*Aspergillus clavatus* NRRL 1	EAW09842.1	21.3%	49.4%	70.0%
PEPTIDE_CLUSTER_388	SNOG_03040	EAT89771.2	dihydroorotase	*Aspergillus terreus* NIH2624	EAU34217.1	68.5%	62.6%	73.6%
PEPTIDE_CLUSTER_546	SNOG_04344	EAT88104.2	pentatricopeptide repeat protein	*Aspergillus clavatus* NRRL 1	EAW15104.1	50.9%	25.0%	47.0%
PEPTIDE_CLUSTER_790	SNOG_06563	EAT86394.2	universal stress protein family domain-containing protein	*Microsporum canis* CBS 113480	EEQ31486.1	39.1%	33.0%	46.4%
PEPTIDE_CLUSTER_971	SNOG_08449	EAT84725.2	pre-mRNA splicing factor	*Botryotinia fuckeliana B05.10*	EDN29475.1	19.5%	91.7%	98.3%
PEPTIDE_CLUSTER_1220	SNOG_10328, SNOG_20109	EAT82663.2, EDP89930.1	C6 transcription factor AlcR	*Pyrenophora tritici-repentis* Pt-1C-BFP	EDU48415.1	43.6%, 17.9%	45.8%, 65.5%	61.6%, 82.7%
PEPTIDE_CLUSTER_1380	SNOG_12033	EAT80445.2	predicted protein	*Pyrenophora tritici-repentis* Pt-1C-BFP	EDU49235.1	31.3%	43.3%	61.0%
PEPTIDE_CLUSTER_1509	SNOG_13350, SNOG_13351	EAT79234.2, EAT79235.2	conserved hypothetical protein	*Pyrenophora tritici-repentis* Pt-1C-BFP	EDU48078.1	26.9%, 71.1%	31.0%, 30.5%	42.6%, 46.9%

(B) New genes

Peptide Cluster	Best informative blastx hit	Organism	Accession	% Hit aligned	% Identity	% Similarity
PEPTIDE_CLUSTER_988 PEPTIDE_CLUSTER_1512 PEPTIDE_CLUSTER_1839	phenylalanine ammonia-lyase, putative	*Aspergillus flavus* NRRL3357	EED51807.1	22.4%	55.3%	68.6%

(C) Unassembled Read-Contigs

Sequence	Best informative blastx hit	Organism	Accession	Hit Length (aa)	% Hit aligned	% Identity	% Similarity
Contig1	putative transposase	*Magnaporthe grisea*	AAK01300.1	556	18.3%	35.3%	58.8%
Contig2	gag-pol polyprotein	*Podospora anserina*	ACD86393.1	2603	6.0%	29.0%	52.3%
Contig26	conserved hypothetical protein	*Pyrenophora tritici-*	EDU48200.1	444	60.1%	45.8%	60.9%

		repentis Pt-1C-BFP					
Contig27	putative transposase	*Phaeosphaeria nodorum*	CAD32687.1	550	65.6%	51.8%	68.4%
Contig34	putative transposase	*Phaeosphaeria nodorum*	CAD32687.1	550	35.8%	43.7%	56.8%
Contig54	hypothetical protein SS1G_11576	*Sclerotinia sclerotiorum* 1980	EDN95697.1	551	25.8%	29.6%	52.1%
Contig90	gag/polymerase/env polyprotein, putative	*Talaromyces stipitatus* ATCC 10500	EED12060.1	461	26.7%	43.1%	65.9%
Contig91	putative transposase	*Phaeosphaeria nodorum*	CAD32689.1	546	35.5%	38.3%	58.2%
Contig121	putative transposase	*Phaeosphaeria nodorum*	CAD32689.1	546	85.3%	43.8%	61.8%
Contig213	hypothetical protein ACLA_028940	*Aspergillus clavatus* NRRL 1	EAW08168.1	113	70.8%	61.3%	68.8%
Contig214	beta-N-acetylhexosaminidase	*Pyrenophora tritici-repentis* Pt-1C-BFP	EDU43007.1	775	22.2%	58.7%	73.8%
Contig303	predicted protein	*Lodderomyces elongisporus* NRRL YB-4239	EDK45775.1	603	33.0%	94.5%	96.5%
Contig463	protein kinase regulator Ste50	*Neosartorya fischeri* NRRL 181	EAW19283.1	487	39.6%	45.2%	54.8%
Contig540	fungal specific transcription factor, putative	*Talaromyces stipitatus* ATCC 10500	EED23703.1	1044	5.4%	77.1%	91.4%
Contig552	phosphatidyl synthase	*Aspergillus flavus* NRRL3357	EED51299.1	267	16.5%	64.4%	66.7%
Contig582	cytochrome oxidase subunit 3; NADH dehydrogenase subunit 2	*Phaeosphaeria nodorum* SN15 (mitochondrial genome)	ABU49451.1; ABU49450.1	275; 572	73.8%, 32.9%	97.0%, 98.4%	97.0%, 98.4%
Contig587	hypothetical protein PTRG_08559	*Pyrenophora tritici-repentis* Pt-1C-BFP	EDU51478.1	288	25.3%	87.7%	94.5%
Contig589	putative senescence-associated protein	*Pisum sativum*	BAB33421.1	282	98.2%	53.9%	60.2%
Contig604	60S ribosomal protein L3	*Pyrenophora tritici-repentis* Pt-1C-BFP	EDU51030.1	392	67.9%	94.7%	97.7%
Contig609	glucose-repressible gene protein	*Botryotinia fuckeliana* B05.10	EDN32782.1	71	95.8%	50.7%	67.6%
Contig610	SAM binding motif containing	*Pyrenophora tritici-*	EDU49259.1	450	62.4%	61.4%	70.7%

	protein	*repentis* Pt-1C-BFP					
Contig633	cytoplasmic dynein heavy chain	*Candida albicans* SC5314	EAL04347.1	4161	5.3%	31.5%	57.2%
Contig753	Set1C component SDC1 (Suppressor of CDC25 protein 1)	*Pichia stipitis* CBS 6054	ABN66789.1	163	31.3%	72.5%	96.1%
Contig754	SEC7-family member, endocytosis and vacuole integrity protein, putative	*Candida dubliniensis* CD36	CAX45574.1	1647	14.1%	50.2%	70.4%
Contig786	potential mitochondrial zinc finger protein Fmp28	*Candida albicans* SC5314	EAK93306.1	184	80.4%	44.4%	60.3%
Contig795	hypothetical protein LELG_05797	*Lodderomyces elongisporus* NRRL YB-4239	EDK47616.1	1527	69.2%	91.4%	93.3%
Contig873	conserved hypothetical protein	*Pyrenophora tritici-repentis* Pt-1C-BFP	EDU50992.1	569	30.4%	68.0%	76.0%
Contig912	conserved hypothetical protein	*Talaromyces stipitatus* ATCC 10500	EED18953.1	551	27.9%	33.1%	63.0%
Contig913	Senescence-associated protein	*Brugia malayi*	EDP31077.1	121	86.8%	50.0%	61.7%

(D) Unassembled Read-Singletons

Sequence	Best informative blastx hit	Organism	Accession	Hit Length (aa)	% Hit aligned	% Identity	% Similarity
G707P5245RH3.T0	vacuolar protein-sorting-associated protein 46	*Pyrenophora tritici-repentis* Pt-1C-BFP	EDU51088.1	197	39.6%	93.6%	93.6%
G707P5441RC9.T0	NADH dehydrogenase subunit 5	*Candida albicans* SC5314	AAG59597.2	552	19.9%	67.3%	81.8%
G707P5444RF3.T0	U-box domain containing protein	*Pyrenophora tritici-repentis* Pt-1C-BFP	EDU44320.1	308	40.3%	28.1%	45.9%
G707P5447FE8.T0	ring finger protein, putative; transcription factor, putative; transcriptional repressor, putative	*Candida dubliniensis* CD36	CAX39756.1	972	21.4%	52.0%	71.5%
G707P61053FG3.T0	hypothetical protein CHGG_09580	*Chaetomium globosum* CBS 148.51	EAQ85566.1	1176	15.6%	26.6%	55.4%
G707P61313FA1.T0	SH3 domain protein	*Aspergillus clavatus* NRRL 1	EAW09601.1	979	23.6%	24.0%	36.1%
G707P61325RA9.T0	conserved hypothetical protein	*Pyrenophora tritici-repentis* Pt-1C-BFP	EDU48266.1	403	17.4%	47.1%	62.9%

G707P6228FC12.T0	Ras GTPase activating protein, putative	*Aspergillus fumigatus* A1163	EDP54022.1	1750	5.7%	35.0%	48.0%
G707P6808FB9.T0	alpha-N-arabinofuranosidase	*Pyrenophora tritici-repentis* Pt-1C-BFP	EDU47783.1	496	17.3%	32.5%	42.5%
G707P8103RA8.T0	telomere-associated RecQ helicase	*Aspergillus fumigatus* Af293	EAL89304.1	352	38.9%	26.3%	49.6%
G707P8157RA9.T0	coproporphyrinogen III oxidase	*Pyrenophora tritici-repentis* Pt-1C-BFP	EDU46533.1	427	20.6%	59.1%	73.9%
G707P8201RA6.T0	rRNA intron-encoded homing endonuclease	*Oryza sativa*	AAK13589.1	66	80.3%	59.3%	70.4%
G707P8216RB4.T0							
G707P846FB1.T0							
G707P886FD7.T0	predicted protein	*Pyrenophora tritici-repentis* Pt-1C-BFP	EDU42002.1	648	14.0%	40.7%	48.4%
