# Supplementary material for: Deep proteogenomics; high throughput gene validation by multidimensional liquid chromatography and mass spectrometry of proteins from the fungal wheat pathogen Stagonospora nodorum
Source: BMC Bioinformatics. 2009 Sep 22;10:301. doi: 10.1186/1471-2105-10-301 (PMC2753851; doi:10.1186/1471-2105-10-301)
Supplement: Additional file 3 — Summary of gene ontology (GO) terms over and under represented in peptide supported S. nodorum genes relative to a random sampling of the whole genome of S. nodorum. Significance of representation was determined via Fisher's exact test, subject to a p-value threshold of 0.05. [file 1471-2105-10-301-S3.PDF]

Additional file 3: Summary of gene ontology (GO) terms over and under represented in peptide supported *S. nodorum* genes relative to a random sampling of the whole genome of *S. nodorum*. Significance of representation was determined via Fisher's exact test, subject to a p-value threshold of 0.05.

OVER-REPRESENTED

Go ID	GO Name	GO level	Genes (peptide supported)	Genes (expected)
**Biological Process**				
GO:0006412	translation	6	75	51
GO:0016068	type I hypersensitivity	7	12	5
GO:0002526	acute inflammatory response	5	12	5
GO:0008652	amino acid biosynthetic process	6	55	39
GO:0006099	tricarboxylic acid cycle	7	16	8
GO:0006007	glucose catabolic process	7	19	10
GO:0006096	glycolysis	5	16	8
GO:0006807	nitrogen compound metabolic process	2	98	84
GO:0043623	cellular protein complex assembly	6	12	6
GO:0051603	proteolysis involved in cellular protein catabolic process	7	17	10
GO:0006511	ubiquitin-dependent protein catabolic process	7	17	10
GO:0009067	aspartate family amino acid biosynthetic process	7	13	7
GO:0042221	response to chemical stimulus	2	25	17
**Cellular Component**				
GO:0005829	cytosol	4	30	13
GO:0005840	ribosome	3	54	35
GO:0043232	intracellular non-membrane-bounded organelle	4	68	49
GO:0044444	cytoplasmic part	3	218	190
GO:0005737	cytoplasm	3	46	32
GO:0033279	ribosomal subunit	3	17	9
GO:0005839	proteasome core complex	3	12	6
GO:0030529	ribonucleoprotein complex	3	92	77
**Molecular Function**				
GO:0003735	structural constituent of ribosome	2	70	44
GO:0003743	translation initiation factor activity	4	22	13
GO:0003743	translation initiation factor activity	3	22	13
GO:0004298	threonine-type endopeptidase activity	6	12	6
GO:0008092	cytoskeletal protein binding	3	9	4
GO:0005524	ATP binding	6	153	137
GO:0016861	intramolecular oxidoreductase activity, interconverting aldoses and ketoses	4	7	3

UNDER-REPRESENTED

**Biological Process**				
GO:0008643	carbohydrate transport	3	4	23
GO:0006355	regulation of transcription, DNA-dependent	7	29	61
GO:0006350	transcription	5	14	39
GO:0006865	amino acid transport	4	2	13
GO:0007047	cell wall organization	4	3	12
GO:0006812	cation transport	4	20	34
GO:0006281	DNA repair	6	13	24
GO:0030001	metal ion transport	5	4	12
GO:0016310	phosphorylation	5	29	41
GO:0006820	anion transport	4	1	6
GO:0009063	amino acid catabolic process	6	1	6
GO:0006260	DNA replication	6	3	9
GO:0045493	xylan catabolic process	7	3	9
GO:0016071	mRNA metabolic process	6	14	22
GO:0008380	RNA splicing	7	8	15

GO:0043412	biopolymer modification	4	54	64
GO:0006629	lipid metabolic process	3	33	42
**Cellular Component**				
GO:0016021	integral to membrane	4	40	163
GO:0005634	nucleus	5	68	114
GO:0005743	mitochondrial inner membrane	4	2	11
GO:0044429	mitochondrial part	4	12	21
GO:0005626	insoluble fraction	3	1	6
**Molecular Function**				
GO:0022804	active transmembrane transporter activity	3	27	77
GO:0015295	solute:hydrogen symporter activity	7	4	25
GO:0005351	sugar:hydrogen symporter activity	7	4	25
GO:0005402	cation:sugar symporter activity	7	4	25
GO:0043565	sequence-specific DNA binding	4	2	19
GO:0008270	zinc ion binding	5	40	74
GO:0003700	transcription factor activity	3	13	34
GO:0004553	hydrolase activity, hydrolyzing O-glycosyl compounds	4	32	58
GO:0005275	amine transmembrane transporter activity	4	2	14
GO:0004497	monooxygenase activity	3	5	19
GO:0008168	methyltransferase activity	4	16	35
GO:0030247	polysaccharide binding	3	3	12
GO:0004871	signal transducer activity	2	14	26
GO:0004672	protein kinase activity	5	32	44
GO:0015297	antiporter activity	5	2	8
GO:0004527	exonuclease activity	5	1	6
GO:0046873	metal ion transmembrane transporter activity	5	3	9
GO:0003887	DNA-directed DNA polymerase activity	6	0	4
GO:0015103	inorganic anion transmembrane transporter activity	6	0	4
GO:0046943	carboxylic acid transmembrane transporter activity	5	0	4
GO:0016701	oxidoreductase activity, acting on single donors with incorporation of molecular oxygen	3	4	10
