# Supplementary material for: Deep proteogenomics; high throughput gene validation by multidimensional liquid chromatography and mass spectrometry of proteins from the fungal wheat pathogen Stagonospora nodorum
Source: BMC Bioinformatics. 2009 Sep 22;10:301. doi: 10.1186/1471-2105-10-301 (PMC2753851; doi:10.1186/1471-2105-10-301)
Supplement: Additional file 4 — Summary of gene ontology (GO) terms over and under represented in peptide supported S. nodorum genes relative to genes supported by EST alignments. Significance of representation was determined via Fisher's exact test, subject to a p-value threshold of 0.05. [file 1471-2105-10-301-S4.PDF]

Additional file 4: Summary of gene ontology (GO) terms over and under represented in peptide supported *S. nodorum* genes relative to genes supported by EST alignments. Significance of representation was determined via Fisher's exact test, subject to a p-value threshold of 0.05.

OVER-REPRESENTED

Go ID	GO Name	GO level	Genes (peptide supported)	Genes (expected)	Total genes
**Biological Process**					
GO:0009073	aromatic amino acid family biosynthetic process	6	9	2	15
GO:0046417	chorismate metabolic process	6	9	2	15
GO:0006399	tRNA metabolic process	7	23	11	49
GO:0006418	tRNA aminoacylation for protein translation	8	19	9	37
GO:0051276	chromosome organization	4	18	9	33
GO:0007018	microtubule-based movement	4	9	3	16
GO:0030705	cytoskeleton-dependent intracellular transport	4	9	3	16
GO:0009067	aspartate family amino acid biosynthetic process	7	13	6	18
GO:0007242	intracellular signaling cascade	4	28	17	59
GO:0051603	proteolysis involved in cellular protein catabolic process	7	17	9	25
GO:0006511	ubiquitin-dependent protein catabolic process	7	17	9	25
GO:0006979	response to oxidative stress	3	7	2	10
GO:0009070	serine family amino acid biosynthetic process	7	10	4	21
**Cellular Component**					
GO:0005829	cytosol	4	30	15	34
GO:0005737	cytoplasm	3	46	26	82
GO:0044430	cytoskeletal part	4	20	8	36
GO:0005839	proteasome core complex	3	12	5	14
GO:0005874	microtubule	4	11	4	20
GO:0005875	microtubule associated complex	4	6	1	12
GO:0043231	intracellular membrane-bounded organelle	3	143	136	496
**Molecular Function**					
GO:0005524	ATP binding	6	153	82	347
GO:0003676	nucleic acid binding	2	162	124	560
GO:0048037	cofactor binding	2	46	28	103
GO:0004812	aminoacyl-tRNA ligase activity	5	20	9	38
GO:0015926	glucosidase activity	5	7	1	12
GO:0004298	threonine-type endopeptidase activity	6	12	5	14
GO:0016836	hydro-lyase activity	4	17	8	29
GO:0003777	microtubule motor activity	2	6	1	12
GO:0008026	ATP-dependent helicase activity	9	15	7	35
GO:0004672	protein kinase activity	5	32	20	111
GO:0005515	protein binding	2	91	69	237
GO:0016614	oxidoreductase activity, acting on CH-OH group of donors	3	41	27	87
GO:0046872	metal ion binding	3	145	140	554
GO:0003735	structural constituent of ribosome	2	70	63	111
GO:0016860	intramolecular oxidoreductase activity	3	11	5	13
GO:0004571	mannosyl-oligosaccharide 1,2-alpha-mannosidase activity	7	3	0	3

UNDER-REPRESENTED

**Biological Process**					
GO:0044247	cellular polysaccharide catabolic process	6	5	14	45
GO:0046942	carboxylic acid transport	4	2	8	36
GO:0006857	oligopeptide transport	4	0	3	4
GO:0006915	apoptosis	4	1	4	7
GO:0010382	cell wall metabolic process	4	3	8	29

GO:0007047	cell wall organization	5	3	8	30
GO:0010556	regulation of macromolecule biosynthetic process	5	37	43	190
GO:0010468	regulation of gene expression	4	37	43	190
**Cellular Component**					
GO:0016021	integral to membrane	4	40	77	414
GO:0005743	mitochondrial inner membrane	4	2	11	28
GO:0005739	mitochondrion	4	45	47	102
**Molecular Function**					
GO:0008137	NADH dehydrogenase (ubiquinone) activity	6	2	9	14
GO:0008324	cation transmembrane transporter activity	5	28	36	159
GO:0004842	ubiquitin-protein ligase activity	6	7	12	29
GO:0005506	iron ion binding	5	32	36	99
